# Effects of Hormonal Contraception Use on Cognitive Functions in Patients With Bulimia Nervosa

**DOI:** 10.3389/fpsyt.2021.658182

**Published:** 2021-05-17

**Authors:** Benedicte Nobile, Laurent Maimoun, Isabelle Danielle Jaussent, Maude Seneque, Kathlyne Dupuis-Maurin, Patrick Lefebvre, Phillippe Courtet, Eric Renard, Sebastien Guillaume

**Affiliations:** ^1^Department of Emergency Psychiatry and Post-Acute Care, CHRU Montpellier, Montpellier, France; ^2^Institute of Functional Genomics, University of Montpellier, CNRS, INSERM, Montpellier, France; ^3^FondaMental Foundation, Créteil, France; ^4^Department of Nuclear Medicine, CHRU Montpellier, Montpellier, France; ^5^INSERM U1046, UMR9214 CNRS, Physiology and Experimental Medicine of the Heart and Muscles, University of Montpellier, CHRU Montpellier, Montpellier, France; ^6^INM, University of Montpellier, INSERM, Montpellier, France; ^7^Department of Endocrinology, Diabetes, and Nutrition, CHRU Montpellier, Montpellier, France; ^8^UMR CNRS 5203, INSERM U1191, Institute of Functional Genomics, University of Montpellier, Montpellier, France

**Keywords:** eating disorders, sustained attention, decision making, hormonal contraception, sexual hormone

## Abstract

**Background:** Growing evidences indicate that sex hormones have an effect on cognitive functions, and that Bulimia Nervosa (BN) is associated with cognitive impairment. The aim of this study was to determine the effect of hormonal contraception (HC) use on four cognitive functions that are impaired in patients with BN.

**Methods:** This retrospective exploratory study included 103 women with a diagnosis of BN based on the DSM-5 criteria. Their age ranged from 15 to 45 years, and 46.6% were taking HC (oral, transdermal, or intrauterine). Cognition was assessed with the d2 test (attention), Iowa gambling task (IGT; decision making), Brixton spatial anticipation test (set shifting), and Rey-Osterrieth complex figure test (central coherence). Data were analyzed with logistic regression models to estimate the adjusted odds ratios (OR) and 95% confidence intervals (CI) of HC effect on the neuropsychological test scores.

**Results:** In the multivariate model, HC use was significantly associated with better scores for two d2 test indices: F-score [OR = 0.98, 95% CI = (0.95; 0.99)] and final total score ratio [OR = 0.87, 95% CI = (0.77; 0.99)]. HC was also associated with a better understanding of the IGT explicit rules. No difference between the two groups (HC and non-HC use) was detected for set shifting and central coherence.

**Conclusions:** This exploratory study suggests that HC could have effects on the sustained attention and concentration in women with BN. More studies are needed to confirm these results.

## Introduction

Bulimia Nervosa (BN) is a severe psychiatric disorder with a lifetime prevalence of about 3% in women (1), high burden, and poor health-related quality of life (2). Despite some evidence-based treatments, the prognosis remains unsatisfactory and more research is needed on BN pathophysiology to find therapeutic targets. BN has been associated with possible endocrine deregulations (3, 4), and also with cognitive impairment, especially in four cognitive functions: decision making, central coherence, set shifting, and attentional networks. Indeed, a meta-analysis concluded that decision making is significantly altered in patients with BN (5). Moreover, a recent meta-analysis found that people with BN have central coherence and set shifting deficiencies that do not significantly differ from those observed in people with Anorexia Nervosa (AN) (6). Attentional networks are also altered in patients with BN (7, 8). Although there are some heterogeneities among studies on cognitive functions in patients with eating disorders (9), a meta-analysis found that studies on patients with BN are more homogenous than those on patients with AN, and that overall, cognitive functions are altered in patients with BN (10). It is important to focus on these impairments because they might contribute to the progression of BN and a poor prognosis (5). Moreover, people with BN might benefit from adjunctive approaches to address these deficiencies, such as cognitive remediation therapy, which has shown positive results in this population (6, 11, 12).

Interestingly, a growing body of evidence suggests that sex hormones (estrogen and progesterone) influence cognitive functions and therefore, these hormones have become the focus of considerable attention in psychiatry (13). First, the two estrogen receptors (ER), ERα and ERβ, are expressed in several brain areas implicated in cognitive functions (e.g., prefrontal cortex, dorsal striatum, nucleus accumbens, hippocampus, and thalamus), suggesting that they might influence their activity (14, 15). Moreover, studies on ovariectomized and menopausal women have shown a significantly higher cognitive function decline in women without hormone replacement therapy (HRT) than in those receiving HRT (16–18). However, another study found that HRT could be deleterious when introduced too late after menopause or ovariectomy (17). Similarly, other studies found deleterious effects of estrogens on cognitive functions in women, and findings remain inconclusive and contradictory (19–21). On the other hand, Egan and Gleason (22) reported that hormonal contraception (HC) users have better visuospatial ability and cognitive flexibility scores than non-users, and these results were HC duration-dependent. Furthermore, low estrogen levels could promote inflammation (increase of IL-6, IL-1, and TNFα) (16), which negatively affects cognitive functions (23). In addition, a recent randomized controlled trial in oligomenorrheic/amenorrheic athletes (who present symptoms similar to those of patients with eating disorders) showed a significant improvement in verbal memory and executive control in participants taking the estrogen replacement compared with untreated women (24).

Second, few studies have investigated the impact of progesterone on cognitive functions. Like ERs, progesterone receptors are expressed in several brain areas implicated in cognitive functions, such as the hypothalamus, thalamus, frontal cortex, and amygdala (25). Moreover, progesterone seems to have a neuroprotective effect by acting on neurotrophic factors, such as brain derived neurotrophic factor (26, 27). Finally, some studies suggested a positive effect of progesterone on the cognitive performances in women (28, 29).

As HC (estro-progestin or progestin alone) provides a constant rate of sex hormones to avoid the hormonal triggers of ovulation, we hypothesized that HC might have an impact on the cognitive functions in patients with BN. To test this hypothesis, we performed an exploratory study in women with BN, who were and were not taking HC, using four neuropsychological tests that evaluate the cognitive functions known to be impaired in BN: the d2 test (attention and concentration), the Iowa gambling task (IGT; decision making), the Brixton spatial anticipation test (set shifting), and the Rey-Osterrieth complex figure test (ROCFT; central coherence).

## Materials and Methods

### Participants

This is a retrospective observational and monocentric study. Participants were consecutively recruited in an outpatient eating disorder unit of the University Hospital Center of Montpellier, France, between 2012 and 2018. Overall, 103 female patients with BN, as defined by the DSM5 criteria, were included. This ancillary study to a larger project was approved by the local ethics committee (CPP Sud Méditerranée IV), and the signed informed consent was obtained from all participants (and from the parents for underage participants). Research was performed according to the tenets of the Declaration of Helsinki.

The inclusion criteria were: female, non-menopausal, between 15 and 45 years of age, French speaking, and with BN according to the DSM5 criteria. In addition, all HC types were allowed (transdermal, hormonal intrauterine device, and oral) as well as all types of hormonal substitution (progestin alone and estro-progestin). The non-inclusion criteria were: another eating disorder other than BN, use of a copper intrauterine device, primary amenorrhea, physical condition precluding the study participation, and refusal to participate in the study.

### Clinical Assessment

Patients were evaluated by a multidisciplinary team of psychiatrists, psychologists, and nutritionists. Diagnoses were established by consensus using the best-estimated procedure and medical records and information from relatives, non-standardized clinical assessments by practitioners, and standardized measures with the Mini-International Neuropsychiatric Interview (MINI).

In addition, patients completed self-administered questionnaires to assess the main characteristics of eating disorders (ED). Five characteristics related to the disease severity were evaluated using the four subscales of the Eating Disorder Examination Questionnaire (EDE-Q) and the Functioning Assessment Short Test (FAST). The EDE-Q is a 33-item screening tool to evaluate ED symptoms. It measures disordered eating over a 28-day period and is scored across four subscales that explore the four core clinical dimensions of EDs: eating concern, body shape concern, weight concern, and restraint. Higher scores indicate a more severe ED (30, 31). In this study, the EDE-Q was used to evaluate the pathology severity and not to establish a diagnosis. The FAST is an interview-administered instrument. It comprises of 24 items divided in five areas of functioning: autonomy, occupational functioning, financial issues, interpersonal relationships, and leisure time. All items are rated using a 4-point scale and the total score is obtained by summing the score of each item. Higher scores indicate more severe difficulties (32).

Sociodemographic and clinical data were systematically recorded by a psychiatrist. All possible psychiatric comorbidities (e.g., bipolar disorder and depression) were assessed with the MINI and all current treatments were also recorded.

### Hormonal Contraception

All HC types were allowed. Among the 48 patients of our sample who were taking HC, 33 (68.75%) were using an estro-progestin-based oral contraceptive; three (6.25%), an intrauterine device with progestin; four (8.33%), a transdermal device with progestin; and one (2.08%), a progestin-only oral contraceptive. Seven patients (14.58%) taking an oral contraceptive did not know its composition (estro-progestin or progestin). Only patients who had been taking HC for at least 3 months (duration needed to observe the HC effect on sex hormones) were included. In our sample, the mean duration of HC use was 3.81 (5.37) years.

As this was an ancillary study to a larger project, blood sex hormone levels were not measured and the patients' menstrual status was not known. The objective of our study was only to determine whether HC intake, in a naturalistic sample of patients with BN, had an effect on the cognitive functions before setting up studies with an appropriate design.

### Neuropsychological Assessment

Crystalline intelligence was assessed with the French version of the National Adult Reading Test (NART), which is a validated tool to measure prior intellectual functioning. The NART is a 50-item single word reading test of graded difficulty. NART is based on the observation that the level of reading ability in the normal adult population is linked to the general intellectual level, and that in patients with dementia, oral reading is preserved (until late in the dementia process) (33).

The patients' cognitive functions were assessed using four neuropsychological tests. The d2 Test of Attention is a useful and validated measure of attention and concentration. This is a one-page cancellation test, consisting of 14 lines, each line with 47 “p” and “d” characters. The characters have one to four dashes that are positioned individually or in pairs above and/or below each letter. The target symbol is a “d” with two dashes (above or below or both). Thus, a “p” with one or two dashes and a “d” with one, three, or four dashes are distracters. The participant's task is to cross out as many target symbols as possible, moving from left to right (34, 35). As the d2 test result interpretation is complex, it has various indices. In this study, six indices were used: GZ, KL, F1, F2, F, and the d2 ratio. GZ corresponds to the number of treated items; it is a quantitative “performance index” that measures the basic cognitive working speed. F1 is the number of omission mistakes (a measure of “attention”) and F2 is the number of confusion mistakes (a measure of “impulsivity”). F is the total number of mistakes, a measure of the “selection abilities.” KL is the number of marked target symbols minus the number of confusion mistakes, and is considered a “concentration performance index” (36). Finally, the d2 ratio is the test total score, obtained with the formula: GZ/F^*^100. Low d2 ratios indicate better attention and concentration.

The Iowa gambling task (IGT) is the most widely used test to assess decision-making. Individuals are given $2,000 in game money and they must maximize this sum over 100 trials by taking cards from one of four decks. Decks A and B are advantageous in the short term, but disadvantageous in the long term. Decks C and D are less advantageous than Decks A and B in the short term, but more advantageous in the long term. Therefore, Decks A and B are “disadvantageous,” and Decks C and D are “advantageous.” At the end of the task, the total score is calculated as follows: (Decks C + Decks D) - (Decks A + Decks B); higher scores indicate a better decision making (37). Discussion has focused on the observation that up to the 50th trial, all trials are ambiguous because there is no information indicating which decks are advantageous or disadvantageous. Thus, it is globally considered that the first 50 trials represent the adaptive phase of the test. Then, trials 51–100 are considered less risky because subjects should have learned which cards are safe or risky. Consequently, the second part of the test is considered the real decision-making test (38). Therefore, the IGT scores from the 1st to the 50th trial and from the 51st to the 100th trial were analyzed separately (1–50 and 51–100 scores). Finally, IGT comprehension was scored from 0 to 2 (from worst to best test comprehension) (39). Due to technical complications, only 58 participants performed this task.

The Brixton spatial anticipation test assesses the ability to detect and follow a rule and the flexibility to a changing pattern. The task consists of a series of pages on which there is one filled-in circle (its location changes on each new page) and empty circles. Patients have to predict the position of the filled-in circle on each subsequent page. The total number of errors made during the task is converted to a scaled score. Higher scores indicate a better set shifting (40). This task was analyzed using clinical norms (41).

The ROCFT is widely used to assess the visuospatial constructional ability and visual memory. The patient receives the ROCF stimulus card and he/she must reproduce this figure immediately and again, 30 min later. The scoring system includes location, accuracy, organization, and reproduction time. The coping style was scored on a scale from 1 to 4 (1 is the best score). As not enough patients scored 4 on this test, patients with scores = 3 and 4 were grouped together (42).

### Statistical Analysis

The study population was described using means (sd) for quantitative variables and percentages for qualitative variables. Psychotropic drugs (i.e., anxiolytic, hypnotic, antidepressant, and antipsychotic) were all computed as a unique variable due to the small sample and the treatment heterogeneity. A univariate logistic regression model was used to study the associations between the sociodemographic and clinical data according to HC use. The sociodemographic and clinical variables associated with the outcome (*p* < 0.10) were included in the multivariate logistic regression model to estimate the adjusted odds ratios (ORs) and their 95% confidence intervals (CI) for exposure variables (neuropsychological test scores). A second multivariate logistic regression model was performed by adding two variables: current major depressive episode and lifetime AN. These variables were not associated with the outcome (*p* < 0.10), but due to their known impact on cognitive performances, they were included in a second model to assess a possible interaction. Some variables contained missing data for some patients (<10%). These missing data were treated as missing data (no imputation).

The significance level was set at *p* < 0.05. Analyses were performed with the SPSS statistical software (version 26; IBM SPSS Statistics for Windows. Armonk, NY: IBM Corp).

## Results

### Sample Characteristics

For this study, 103 women with BN were enrolled: 55 (53.4%) did not use HC and 48 (46.6%) used. Their mean age was 27.36 (7.31) years, and their mean body mass index was 21.95 (3.18) kg/m^2^. None of them had primary amenorrhea. In both groups (HC and no HC), ~40% of the patients did not have regular menstrual cycles. The mean BN duration was 8.51 (6.92) years, and the mean age at disease onset was 18.54 (5.24) years. The mean EDE-Q total score was 4.22 (1.10), and the mean FAST total score was 25.54 (1.10), confirming the BN severity and an impaired global functioning.

### Comparison of the Sociodemographic and Clinical Data According to HC Use

Sociodemographic and clinical data were comparable between the two groups (HC and no-HC) (Table 1). Similarly, variables that could influence cognitive performances, such as BN duration, regular menstrual cycle, BN severity, lifetime AN, current major depressive episode, crystalline intelligence, and global functioning (i.e., EDE-Q total score, FAST total score and sub-scores, and NART score), were not significantly different between the two groups. Psychiatric comorbidity rates were comparable between the two groups (Table 1). Conversely, taking psychotropic drugs and having children tended to be more frequent in the participants using HC (*p*-value < 0.10). Consequently, these two parameters were included in the multivariate logistic regression model.

**Table 1:** Association between hormonal contraception and sociodemographic and clinical data.

**Variables**	**Hormonal contraception**				***P*-value**
	**No**		**Yes**		
	***N*** **=** **55**		***N*** **=** **48**		
	***n***	**%**	***n***	**%**	
Age, years^(1)^	27.38 (6.99)		27.34 (7.73)		0.98
Study Level					0.40
<12 years	7	14.6	10	21.3	
≥12 years	41	85.4	37	78.7	
Children					0.08
No	47	85.5	34	70.8	
Yes	8	14.5	14	29.2	
In couple					0.66
No	32	58.2	30	62.5	
Yes	23	41.8	18	37.5	
Working					0.53
No	8	14.5	5	10.4	
Yes	47	85.5	43	89.6	
Body mass index, kg/m^2^^(1)^	21.68 (3.07)		22.27 (3.31)		0.35
Illness duration, years^(1)^	7.91 (6.90)		9.15 (7.01)		0.51
Regular menstrual cycles					0.53
No	17	35.4	18	41.9	
Yes	31	64.6	25	58.1	
AN lifetime					0.39
No	37	67.3	36	75	
Yes	18	32.7	12	25	
NART Score^(1)^	21.33 (3.68)		21.61 (4.35)		0.73
EDE-Q Total Score^(1)^	4.20 (1.20)		4.24 (1.01)		0.86
FAST - Autonomy^(1)^	3.41 (2.95)		3.31 (2.62)		0.87
FAST - Professional activity^(1)^	4.61 (4.79)		4.28 (4.79)		0.76
FAST - Cognitive^(1)^	6.20 (3.92)		5.22 (3.93)		0.22
FAST - Finances^(1)^	2.60 (2.20)		2.17 (2.22)		0.33
FAST - Relationship^(1)^	8.37 (4.82)		7.17 (4.63)		0.22
FAST - Hobbies^(1)^	2.00 (1.91)		2.00 (1.82)		1.00
FAST - Total Score^(1)^	26 (14.73)		25.11 (16.32)		0.81
Psychotropic drugs					0.08
No	37	71.2	26	54.2	
Yes	15	28.8	22	45.8	
Current major depressive episode					0.16
No	36	67.9	37	80.4	
Yes	17	32.1	9	19.6	
Bipolar disorder					NA
No	49	92.5	47	100.0	
Yes	4	7.5	0	0	
Anxiety disorders					0.24
No	23	43.4	26	55.3	
Yes	30	56.6	21	44.7	

(1) *Continuous variables were expressed as mean (sd)*.

### Effects of HC on Neuropsychological Test Scores

For the d2 test, no significant association was detected by univariate analysis (Model 0, Table 2). Conversely, in the multivariate analysis adjusted for potential confounders, three d2 indices were significantly associated with HC intake (Model 1 and Model 2, Table 2). Specifically, the F-score and the d2 ratio were significantly associated with HC use in both multivariate models [e.g., F: OR = 0.98, 95% CI = (0.95; 0.99), *p*-value = 0.04, Model 1; and d2 ratio: OR = 0.87, 95% CI = (0.77; 0.99), *p*-value = 0.03, Model 1] (Table 2). Moreover, the F1 score tended to be associated with HC intake in Model 1 [OR = 0.98, 95% CI = (0.96; 1.0), *p*-value = 0.07] and was significantly associated with HC in Model 2 [OR = 0.97, 95% CI = (0.94; 0.99), *p*-value = 0.03] (Table 2).

**Table 2:** Association between the neuropsychological test scores and hormonal contraception use.

**Variable**	**Hormonal contraception**				**Model 0**		**Model 1**		**Model 2**	
	**No**		**Yes**				
	***N*** **=** **55**		***N*** **=** **48**				
	***n***	**%**	***n***	**%**	**OR [95% CI]**	***P*-value**	**OR [95% CI]**	***P*-value**	**OR [95% CI]**	***P*-value**
d2 GZ^(1)^	481.78 (82.08)		475.57 (79.97)		1.00 [0.99; 1.00]	0.70	1.0 [0.99; 1.01]	0.97	0.99 [0.99; 1.01]	0.77
d2 KL^(1)^	178.26 (41.43)		179 (36.98)		1.00 [0.99; 1.01]	0.93	1.0 [0.99; 1.02]	0.48	1.00 [0.99; 1.02]	0.59
d2 F1^(1)^	25.26 (25.02)		19.21 (15.47)		0.99 [0.96; 1.01]	0.17	0.98 [0.96; 1.00]	0.07	0.97 [0.94; 0.99]	**0.03**
d2 F2^(1)^	2.15 (4.97)		0.81 (1.19)		0.83 [0.66; 1.05]	0.12	0.82 [0.64; 1.05]	0.11	0.87 [0.70; 1.09]	0.22
d2 F^(1)^	27.41 (25.98)		20.02 (15.73)		0.98 [0.96; 1.00]	0.11	0.98 [0.95; 0.99]	**0.04**	0.97 [0.94; 0.99]	**0.02**
Ratio D2^(1)^	5.60 (4.95)		4.16 (3.00)		0.91 [0.81; 1.02]	0.10	0.87 [0.77; 0.99]	**0.03**	0.84 [0.72; 0.97]	**0.02**
IGT - Total Score^(1)^	7.85 (30.63)		15.90 (33.01)		1.01 [0.99; 1.02]	0.28	1.01 [0.99; 1.02]	0.36	1.00 [0.99; 1.02]	0.71
IGT - Score 1–50^(1)^	0.76 (11.38)		1.07 (14.64)		1.00 [0.97; 1.04]	0.92	0.99 [0.96; 1.04]	0.94	0.99 [0.95; 1.03]	0.62
IGT - Score 51–100^(1)^	7.09 (21.37)		14.83 (22.34)		1.02 [0.99; 1.04]	0.13	1.02 [0.99; 1.04]	0.17	1.01 [0.99; 1.04]	0.41
IGT comprehension						**0.02**		**0.01**		**0.02**
None or partial	20	69.0	11	37.9	1		1		1	
Full	9	31.0	18	62.1	3.64 [1.23; 10.78]		4.55 [1.41; 14.66]		4.20 [1.24; 14.24]	
Brixton Score						0.43		0.20		0.34
<7	29	54.7	22	46.8	1		1		1	
≥7	24	45.3	25	53.2	1.37 [0.62; 3.02]		1.77 [0.74; 4.21]		1.56 [0.62; 3.90]	
ROCFT - Style of coping						0.56		0.26		0.15
1	27	51.9	25	54.3	1		1		1	
2	18	34.6	12	26.1	0.72 [0.29; 1.79]		0.67 [0.26; 1.74]		0.71 [0.26; 1.97]	
≥3	7	13.5	9	19.6	1.39 [0.45; 4.29]		2.14 [0.60; 7.66]		3.25 [0.80; 13.18]	

(1) *Continuous variables were expressed as mean (sd)*.

*Model 0: Crude association*.

*Model 1: Adjusted for having children and using psychotropic medicines*.

*Model 2: Adjusted for having children, using psychotropic medicines, current major depressive episode, and lifetime AN. Bold values represent significant results (p-value < 0.05)*.

The IGT scores (total score, scores 1–50 and 51–100) were not associated with HC use in both univariate and multivariate analyses. However, the IGT comprehension score was associated with HC use [OR = 4.55, 95% CI = (1.41; 14.66), *p*-value = 0.01, Model 1, Table 2], with a better comprehension in the HC group. The large CI values (from 1.4 to 14.6) could be explained by the small number of participants who performed this test (56.3% of the sample). Nevertheless, 62% of the patients with HC displayed a full comprehension of the IGT compared with 31% in the non-HC group.

No significant between-group difference was observed concerning the Brixton scores or style of coping for the ROCFT.

## Discussion

This proof-of-concept observational study is the first, to our knowledge, to assess neuropsychological performance in a large population of women with BN according to HC use. We found that patients with BN taking HC had a better total score (ratio d2), made fewer mistakes (F and F1 scores) in the d2 test, and had a better comprehension of the IGT compared with the patients who were not taking HC when the analysis was adjusted for potential confounders. These results suggest that the selection abilities (F-score) and global sustained attention (ratio d2 score) are better in patients with BN taking HC. Finally, HC users seem to better understand the IGT. Interestingly, results became significant when adjusted for potential confounders, particularly the clinical variables that might influence cognitive performances. Although current major depressive episode and lifetime AN were not statistically different between the HC and non-HC groups, they are known to have a negative impact on the cognitive functions. For instance, patients with BN and history of lifetime AN have a poorer cognitive functioning (43, 44), and patients with depression have cognitive impairments (45). Therefore, after the addition of these two variables in the second multivariate model, some of the d2 scores and IGT comprehension were significantly different between the two groups. This suggests a possible important association between HC use and cognitive performance in patients with BN.

This study presents several strengths. First, the quite large sample was consecutively recruited in a specialized unit, and represented a real-life population of patients with BN. Then, a large set of validated tools was used to assess cognition. Furthermore, many confounding factors known to affect cognitive performances were tested (e.g., menstrual cycle irregularity, previous history of AN, psychiatric comorbidities, illness duration, daily functioning), and the two multivariate models took into account the two variables that were most strongly associated with HC use (having children and psychotropic medications) and the two variables known to be associated with cognitive performances (current depressive episode and lifetime AN). Finally, targeting patients with BN allowed the assessment of HC influence on the cognitive performances in patients with potential neuropsychological weaknesses, and thus, to investigate HC impact on the tested functions by avoiding the bias associated with the sex hormone deficiency classically found in AN. This can also explain why our significant results for the d2 test were not statistically very powerful. This could be due to the low HC dosage and/or the relative normality of sex hormone levels in BN.

There are also major limitations. Particularly, the inherent biases of the observational and case-control study design as well as the different HC treatments and doses received by the patients limit the conclusions that can be drawn. Also, the estrogen and progesterone levels and menstrual cycle phases of our patients were not known because of the study design (retrospective, ancillary study). This could be problematic because of the menstrual status influence on cognitive performances (46). However, first, it is quite unlikely that all 55 patients in the non-HC group were in the same menstrual cycle phase (e.g., phase with the lowest or highest progesterone level). Therefore, the non-HC group might represent patients with BN at all menstrual cycle stages. Second, patients in the HC group should have had a constant sex hormone level (i.e., a feature of HC to inhibit ovulation). By limiting the sex hormone changes, HC might reduce the impact of their natural fluctuations on cognitive functions. Third, given the large panel of HC types (intrauterine device, transdermal, oral contraceptives with progestin alone, or with estro-progestin), it was not possible to specifically evaluate the effect of HC type and duration.

Despite these limitations, these results of this exploratory study should be seen as the first step of new investigations with a more appropriate and sophisticated design. For instance, it would be important to quantify the sex hormone levels and their association with cognitive performances as done in the ongoing randomized controlled trial by Paslakis et al. on patients with AN (47). In conclusion, our results suggest a potential interest of HC to improve a sustained attention in women with BN that must be validated by additional studies.

## Data Availability Statement

The raw data supporting the conclusions of this article will be made available by the authors, without undue reservation.

## Ethics Statement

The studies involving human participants were reviewed and approved by CPP Sud Méditerranée IV. Written informed consent to participate in this study was provided by the participants' legal guardian/next of kin.

## Author Contributions

BN: managing the literature searches and wrote the first draft of the manuscript and performed statistical analysis. IJ: performed the statistical analysis, reviewing, and comments of the manuscript. MS, LM, KD-M, PL, and PC recruited patients, reviewing, and comments of the manuscript. SG: recruited patients, designed the study, reviewing, and comments of the manuscript. All authors contributed to and have approved the final manuscript.

## Conflict of Interest

The authors declare interests in relation with one or more organization that could be perceived as a possible conflict of interest in the context of the subject of this manuscript. The relationships are summarized as follows. SG received honoraria or research or educational conference grants from Bristol-Myers Squibb, Otsuka, Servier, Lundbeck, AstraZeneca, and Janssen. PC reports no shares; has paid positions at the University of Montpellier and CHU Montpellier; is on the advisory board at Servier; and has no other involvement. The remaining authors declare that the research was conducted in the absence of any commercial or financial relationships that could be construed as a potential conflict of interest.
